# Mesenchymal Stromal Cells: Potential Option for COVID-19 Treatment

**DOI:** 10.3390/pharmaceutics13091481

**Published:** 2021-09-16

**Authors:** Dragan Primorac, Martin Čemerin, Vid Matišić, Vilim Molnar, Marko Strbad, Lenart Girandon, Lucija Zenić, Miomir Knežević, Stephen Minger, Denis Polančec

**Affiliations:** 1 St. Catherine Specialty Hospital, 10000 Zagreb, Croatia; vid.matisic@svkatarina.hr (V.M.); vilim.molnar@svkatarina.hr (V.M.); 2 Eberly College of Science, The Pennsylvania State University, University Park, State College, PA 16802, USA; 3 The Henry C. Lee College of Criminal Justice and Forensic Sciences, University of New Haven, West Haven, CT 06516, USA; 4 Medical School, University of Split, 21000 Split, Croatia; 5 Faculty of Dental Medicine and Health, Josip Juraj Strossmayer University of Osijek, 31000 Osijek, Croatia; 6 Faculty of Medicine, Josip Juraj Strossmayer University of Osijek, 31000 Osijek, Croatia; 7 Faculty of Medicine, University of Rijeka, 51000 Rijeka, Croatia; 8 Medical School REGIOMED, 96450 Coburg, Germany; 9 School of Medicine, University of Zagreb, 10000 Zagreb, Croatia; martincemerin@gmail.com; 10 Educell Ltd., 1236 Trzin, Slovenia; marko.strbad@biobanka.si (M.S.); lenart.girandon@educell.si (L.G.); miomir.knezevic@educell.si (M.K.); 11 Biobanka Ltd., 1236 Trzin, Slovenia; 12 Srebrnjak Children’s Hospital, 10000 Zagreb, Croatia; lzenic@bolnica-srebrnjak.hr (L.Z.); dpolancec@bolnica-srebrnjak.hr (D.P.); 13 National Institute of Biology, 1000 Ljubljana, Slovenia; stephen.minger@nib.si

**Keywords:** COVID-19, mesenchymal stromal cells, MSCs, immunomodulation, ARDS

## Abstract

The COVID-19 pandemic has significantly impacted the way of life worldwide and continues to bring high mortality rates to at-risk groups. Patients who develop severe COVID-19 pneumonia, often complicated with ARDS, are left with limited treatment options with no targeted therapy currently available. One of the features of COVID-19 is an overaggressive immune reaction that leads to multiorgan failure. Mesenchymal stromal cell (MSC) treatment has been in development for various clinical indications for over a decade, with a safe side effect profile and promising results in preclinical and clinical trials. Therefore, the use of MSCs in COVID-19-induced respiratory failure and ARDS was a logical step in order to find a potential treatment option for the most severe patients. In this review, the main characteristics of MSCs, their proposed mechanism of action in COVID-19 treatment and the effect of this therapy in published case reports and clinical trials are discussed.

## 1. Introduction

During the ongoing coronavirus disease 2019 (COVID-19) pandemic, a wide variety of therapeutic agents have been developed to combat the disease. The use of mesenchymal stromal cells (MSCs) is an emerging therapeutic strategy for the treatment of severely ill patients. MSCs have been studied in numerous medical conditions, and their immunomodulatory and regenerative potential is well documented [1]. MSCs are currently being used for two purposes which do not exclude one another, namely, immunomodulation and tissue regeneration [2,3]. MSCs are being used to treat osteoarthritis, cartilage lesions, perianal fistulas, bone defects, scar tissue reduction, alopecia, chronic wounds, diabetic foot ulcers, etc. [4]. Previous studies on systemic MSC treatment are the cornerstone for treating COVID-19 patients because data show that they lead to a clear improvement in lung function and survival in the setting of ARDS [5]. They are also well-tolerated and do not cause major adverse events, with fever being the only associated risk; therefore MSC-based therapies are currently available for use in critically ill patients [6,7]. In this review, the potential benefits and safety of MSC treatment for COVID-19 patients are highlighted and reviewed in the existing literature on the topic. Furthermore, we summarize relevant knowledge considering the pathophysiological course of COVID-19 to better understand how MSC treatment can help infected patients.

## 2. COVID-19

The current outbreak of COVID-19 was caused by the novel coronavirus officially named severe acute respiratory syndrome coronavirus-2 (SARS-CoV-2). Since SARS-CoV-2 is a new virus that the human population had not been in contact with before, a population-wide lack of effective antibodies is the major problem. Disease course of COVID-19 can be divided into asymptomatic, mild, moderate, severe and critical [8,9]. Mild course presents with symptoms such as fever, cough, sore throat, malaise, headache, muscle pain, nausea, vomiting, diarrhea, loss of taste and smell but without the presence of dyspnea, abnormal chest imaging or shortness of breath. Moderate course is characterized by lower respiratory tract involvement assessed by clinical examination and imaging with oxygen saturation (SpO2) ≥ 94% on room air at sea level. The severe course is characterized with SpO2 < 94% on room air at sea level, a ratio of arterial partial pressure of oxygen to fraction of inspired oxygen (PaO2/FiO2) < 300 mm Hg, respiratory frequency >30 breaths/min, or lung infiltrates in more than 50% of lung parenchyma. Critical course presents with respiratory failure, septic shock and/or multiple organ dysfunction or failure [9,10].

### 2.1. Pathophysiology

The surface of the virus contains projections composed of specific structural proteins named spike (S) proteins [11]. The S1 domain of the protein contains the receptor binding domain which exposes the S2 domain cleavage site by binding to the receptor. The S2 domain is then able to fuse with the membrane of the host cell [12]. Studies have demonstrated a specific, high affinity association between the S1 domain and the angiotensin-converting enzyme 2 (ACE2) protein [13]. Furthermore, with HeLa cells, Zhou et al. demonstrated that SARS-CoV-2 is able to infect only ACE2 expressing cells [14]. This finding suggests that the ACE2 protein may be the main receptor that facilitates the entry of the virus into the cell.

To better understand the effect of SARS-CoV2 on the human body, it is crucial to determine the expression and distribution of ACE2, since it is the route of SARS-CoV2 infection, and the infected organ may depend on the expression and distribution of ACE2 [15]. Studies have demonstrated the broad distribution of ACE2 in various organs, tissues and cell types such as oral mucosa, endothelial cells from small and large arteries and veins and lymphocytes [16,17]. Consistent with the distribution pattern in other organs, the brain only revealed endothelial and smooth muscle cell expression of ACE2 [17]. High expression of ACE2 was found in cardiomyocytes, proximal tubular epithelial cells and bladder urothelial cells, but the most prominent finding was very high level expression of ACE2 in ileal epithelial cells and respiratory tract epithelial cells [18]. In lung tissue, the highest level of ACE2 was found in type II alveolar cells [16,19]. This expression pattern provides a possible explanation for COVID-19 symptoms that primarily affect the lung tissue, but also emphasizes that multiple organs are involved in the pathophysiologic course of the disease [20,21].

### 2.2. Cytokine Storm

Patients with COVID-19 usually present with fever, body aches, breathlessness, malaise, dry cough, sore throat and gastrointestinal issues as nonspecific symptoms [22,23,24]. The clinical condition of patients can deteriorate with pneumonia, which is followed by either recovery or severe disease (acute respiratory distress syndrome (ARDS), acute kidney injury (AKI) and multiorgan failure) [25,26]. The innate immune response is the first line of defense against viral infection. Antigen-presenting cells process viral antigens and present them to natural killer cells and T-cells via major histocompatibility complex (MHC) molecules, thereby activating both innate and adaptive immune responses. However, if the immune response is dysregulated or excessive, it may cause widespread damage to the body [11]. This condition, referred to as “cytokine storm“, often correlates with a more severe clinical course [27,28]. The proposed mechanism for the development of the cytokine storm in COVID-19 includes the capability of SARS-CoV-2 to delay the initial immune response, but also includes the overactivation of the immune system in later stages of viral clearance which compensates for the initial viral clearance failure [29]. Macrophages play a central role in the initial response to the virus. SARS-CoV-2 reduces interferon (IFN) secretion in M1 macrophages and conversely increases the production and secretion of pro-inflammatory cytokines [28,30]. The most important cytokines involved in the cytokine storm response include interleukin 1 (IL-1), IL-6, tumor necrosis factor-alpha (TNF-α), IL-8, IL-18 [29,31]. As the disease progresses, additional macrophages are recruited which, in turn, amplify the inflammatory process. Depending on the tissue microenvironment, macrophages either continue to produce pro-inflammatory cytokines or reprogram toward the M2 phenotype which facilitates the resolution of inflammation [30]. This understanding provides a possible target for therapeutic interventions that specifically target the inflammatory reaction by modulating the tissue microenvironment.

### 2.3. Current Treatment Options

Various pharmacological agents have been used as a potential treatment for COVID-19. Unfortunately, the efficacy and safety of those treatments remain inconclusive, as the results from trials and observational studies are often contradictory [32]. Studies have shown that oral or intravenous corticosteroids exert positive effects on the clinical condition of COVID-19 patients, and that their application is correlated with reduced 28-day mortality [33,34,35]. Conversely, several studies have shown no statistical correlation between the therapeutic effects of corticosteroids in regard to mortality [36,37]. Furthermore, corticosteroids have been associated with a possible risk of side effects including vascular necrosis, diabetes, infection as well as delayed viral clearance [34,36,38,39]. Antiviral agents have also been used to treat COVID-19, with remdesivir being the most promising candidate. Studies have shown conflicting evidence regarding the efficacy of this approach. Some studies have reported either reduced mortality in patients treated with remdesivir or reduced recovery time [33,36,40]. Other studies reported inconclusive data regarding the beneficial effects of remdesivir, suggesting that further, better-controlled studies are necessary to determine the potential beneficial effects of this medication in the treatment of COVID-19 patients [41,42,43]. Nevertheless, phase 3 study results indicated a reduction in hospitalization period, but no effects in terms of mortality when compared to placebo [44]. Convalescent plasma has also been used as a potential treatment for severe cases of COVID-19. Existing studies provide only low-quality evidence to support the benefits of convalescent plasma treatment, but the reported incidence of serious adverse effects was low [45,46]. However, new data support the use of convalescent plasma for early treatment (3 days from symptom onset), since it was shown to help in disease progression [47,48]. Patients with impaired humoral immune response could potentially benefit significantly from convalescent plasma therapy [49].

## 3. Mesenchymal Stromal Cells Treatment

### 3.1. MSC Mechanism of Action

**Figure 1:**
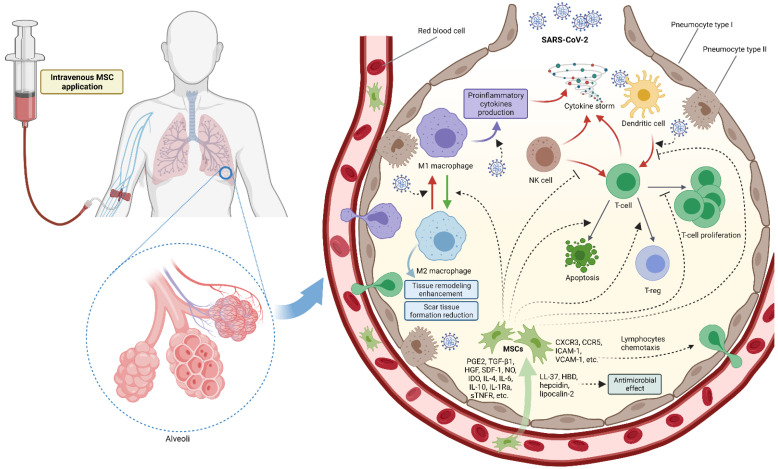
Presumed effect of COVID-19 mesenchymal stem cell therapy. Intravenous administration enables MSCs to travel to the lung microvasculature. MSCs extravasate in the alveoli and are then found in the proinflammatory microenvironment caused by the replication of the virus and subsequent immune response, causing the “cytokine storm”. MSCs are stimulated by the surrounding cytokines and respond by secreting molecules that suppress inflammation, have an antimicrobial effect, cause lymphocyte chemotaxis, stimulate macrophages to change their phenotype from proinflammatory M1 phenotype to anti-inflammatory M2 phenotype. They also inhibit T-cell proliferation and induce T-cell apoptosis and differentiation into T-regulatory cells. MSC–mesenchymal stem cell, PGE2–prostaglandin E2, TGF-β1–transforming growth factor β1, HGF–hepatocyte growth factor, SDF-1–stromal cell-derived factor 1, NO–nitrous oxide, IDO–indoleamine 2,3-dioxygenase, IL–interleukin, IL-1Ra–IL-1 receptor antagonist, sTNFR–soluble tumor necrosis factor α receptor, CXCR3–C-X-C motif chemokine receptor 3, CCR5–C-C motif chemokine receptor 5, ICAM-1–intracellular adhesion molecule 1, VCAM-1–vascular cell adhesion protein 1, LL-37–human cathelicidin antimicrobial peptide, HBD–human beta defensin, NK–natural killer, T-cell–T lymphocyte, T-reg–regulatory T lymphocyte. Created with BioRender.com.

The use of MSCs has emerged as a new treatment option for COVID-19 patients. MSCs have been extensively investigated, but their in vivo origin is yet to be clearly defined. Perivascular localization and expression of some molecular markers indicated that MSCs are multipotent cells derived from pericytes in the microvasculature [50,51,52,53]. This concept proposes that pericytes, when activated by specific stimuli such as injury or inflammation, are released from their association with the basal lamina of the blood vessel. Even though the proposed concept could explain why these cells could be obtained from virtually all vascularized organs and tissues, no clear consensus about their identity has been reached, because the absence of a molecular marker profile, shared exclusively by MSCs and pericytes, precludes a definitive association between these two cell types [53]. When MSCs become activated by the surrounding microenvironment, they interact with the cells of the immune system [52]. Signals that induce this new MSC phenotype are predominantly proinflammatory cytokines including IL-1, IFN-γ, TNF-α, IL-2 and IL-12 [51,54,55]. When found in such a proinflammatory environment, MSCs react by secreting molecules that inhibit the overaggressive reaction of the immune system and establish a stable microenvironment for regenerative processes [52]. These secreted molecules include prostaglandin E2, transforming growth factor β1 (TGF-β1), hepatocyte growth factor (HGF), stromal cell-derived factor 1 (SDF-1), nitrous oxide (NO), indoleamine 2,3-dioxygenase (IDO), IL-4, IL-6, IL-10, IL-1 receptor antagonist (IL-1Ra) and soluble tumor necrosis factor-a receptor (sTNFR) (Figure 1) [55]. Furthermore, MSCs produce a wide variety of chemokines and adhesion molecules including C-X-C motif chemokine receptor 3 (CXCR3), C-C motif chemokine receptor 5 (CCR5), intracellular adhesion molecule 1 (ICAM-1) and vascular cell adhesion protein 1 (VCAM-1), which are necessary for chemotaxis of lymphocytes, ensuring their proximity to MSCs which are then able to exert their optimal suppressive function [51,54]. Studies showed that MSCs potently inhibit T-cell proliferation and induce their apoptosis and differentiation in T-reg cells. T-cells are also indirectly inhibited by the action of MSCs on dendritic cells and natural killer (NK) cells [55]. MSCs also induce M1 macrophages to change their phenotype to the anti-inflammatory M2 phenotype, which further enhances tissue remodeling and reduces scar tissue formation [51,55,56]. Studies have shown that MSCs respond differently depending on environmental stimuli [54]. It seems that MSCs display inhibitory effects on T cells only when there is a strong proinflammatory signal, whereas no inhibitory effect is observed in the absence of an inflammatory signal. Such behavior indicates the plasticity of immunomodulation by MSCs [54,55]. A further beneficial property with regards to COVID-19 is their tendency to gravitate towards the lungs immediately after IV infusion, as they can largely be found in lungs after 24 h, which means they can modulate excessive immune response on-site; they can be traced in vivo after that time point, as they migrate to other organs and tissues, with studies reporting their detectability from 24 h up to 14 days [57,58]. Another potent property of MSCs is their antimicrobial effect exerted by secreting antimicrobial peptides such as LL-37, human β-defensin-2, hepcidin and lipocalin-2 [59]. In light of COVID-19 treatment, this effect is more than welcome, knowing that patients who require mechanical ventilation often complicate with bacterial superinfections which correlate with increased patient mortality and longer hospital stay [60,61].

### 3.2. MSC Markers

Human MSCs are known to constitute a heterogeneous population of cells, and their properties and functionality depend on the environmental characteristics. They differ in morphology, physiology and in the expression of surface antigens. Until now, no single specific marker has been identified to isolate an MSC from tissue samples. Their characteristics are based upon the expression of adhesion molecules, proteins of extracellular matrix, cytokines and growth factor receptors [62,63,64,65]. However, the presence of these markers may change in vitro due to the specific culture conditions and the duration prior to the individual passages [66]. Some antigens may be found on freshly isolated MSCs, but their expression disappears in the culture. Interestingly, such a phenomenon was observed in the case of multilineage progenitor cells (MLPC) which might be a unique population of MSCs that, in fresh isolates, expressed CD34 antigen but were no longer observed in culture [67]. A similar observation was described by Fibbe and co-workers when their group studied MSCs obtained from mouse fetal lungs [68]. Equal results were obtained by observation of chemokine receptors expressed on the cells. However, MSCs also express a wide variety of the marker characteristics for other cell types, [69,70]; nonetheless, CD13, CD29, CD44, CD73 (SH3 and SH4), CD90, CD105 (endoglin or SH2), CD106 (vascular cell adhesion molecule or VCAM-1), CD117, CD166 and CD271 are mostly common to all MSCs, regardless of their source [62,71]. In parallel, MSCs do not possess markers which are typical for hematopoietic and endothelial cell lineages, such as CD11b, CD14, CD31, CD34, CD133 or CD45 (according to the minimum ISCT criteria) [72,73]. In this context, the flow cytometry method has played a significant role in determining the receptor expression and phenotyping of MSCs, unraveling the molecular basis of their in vivo effects and immunomodulatory role. The International Society for Cell and Gene Therapy Mesenchymal Stromal Cell committee issued minimal criteria with which to define the in vitro expanded MSCs as plastic adherent, expressing CD73, CD90 and CD105, lacking the expression of hematopoietic and endothelial markers CD11b, CD14, CD19, CD34, CD45, CD79a and HLA-DR, and capable of in vitro differentiation into adipocyte, chondrocyte and osteoblast lineages. Several issues accompany the concept, especially as MSC characteristics vary depending on the tissue source. Certain percentages (depending on donors and passages) of adipose-derived MSCs are positive for the CD34 marker which, in addition to HLA-DR, seem to be related to culture conditions (plastic adherence, media composition) and cell-isolation methods [74]. A recent work scrutinized native and culture-expanded (10 passages) bone marrow MSCs, determining a range of cell-surface signature markers from immune regulation and proliferation to cell death [75]. Interestingly, the adhesion molecule CD106 (VCAM1), which bestows upon MSCs the ability to modulate T-helper subsets and promote vasculogenesis, was highly expressed in unpassaged MSCs and decreased rapidly in culture. The same held true for death-inducing FasL expression which, via the Fas signaling pathway and regulatory T cells, triggers immunotolerance, while expression of the Fas marker was absent-to-low, yet seemed to confer MSC resistance to autocrine or T-cell mediated apoptosis. Finally, the loss of the CD10 motility marker and the CD71 proliferation marker, together with an increase of the p16 senescence marker, gave an insight into a critical cell-doubling time for producing superior quality MSCs.

All the above demonstrate the need for a standardized MSC culture procedure and strictly phenotype-determined, fine-balanced MSC aimed at COVID-19 treatment. Additionally, MSC-based products can express variable levels of procoagulant tissue factor (CD142) which might compromise beneficial MSC effects in critical COVID-19 patients at high risk for disseminated intravascular coagulation [76]. Therefore, stringent protocols for MSC COVID-19 therapy use are awaited.

### 3.3. Systemic MSC Treatment

Graft-versus-host disease (GvHD) was the first condition to which systemic treatment with MSCs was applied; subsequently, there was an almost exponential growth of experimental clinical use of MSCs in the treatment of GvHD [77]. Systemic treatment required cell cultivation, as the isolation of MCSs from tissue does not yield large enough cell numbers. Due to the limited availability of MSCs in different countries related to the availability of registered MSC products, legislation barriers as well as a lack of certified GMP production sites that can provide sufficient therapeutic doses of MSC for treatment, there was the need to develop MSC treatment regionally [78]. Scaled production might still be a problem in the future, including transportation capabilities and having doses available for patients in time. A need might also arise for establishing point of care facilities within hospitals or close to them to fulfill demand. The results of GvHD treatment, a systemic inflammatory condition, with regards to safety and efficiency encouraged clinicians to use MSCs also for other conditions, with a number of studies being carried out across medical specialties, including chronic obstructive pulmonary disease (COPD), ARDS, emphysema and others [79,80]. With a growing number of inflammatory indications in which MSCs treatment has been used, it became evident that systemic MSC treatment, be it autologous or allogeneic, is safe [6]. Previous preclinical research on MSC application for influenza-induced lung injury found that they prevent or reduced H5N1- and H9N2-associated lung injury in infected mice [81,82]. In 2013, a clinical study explored the use of menstrual blood-derived MSC for treatment of H7N9 influenza-induced ARDS. Seventeen patients were treated with MSC therapy and 44 were enrolled in the control group. The results were promising, as they demonstrated a reliable safety profile (five patients were followed up for four years after treatment) and decreased mortality in the treatment group compared to the controls, 17.6% and 54.5% respectively [81]. Experience and data from previous studies on systemic MSC treatment provide a firm rationale for COVID-19 treatment, since lung damage, ARDS and cytokine storm are commonly observed in severe and critical patients.

## 4. Review of Available Studies

To date, several clinical trials and case report studies have been conducted to determine the safety and efficacy of MSC application in COVID-19 patients. The reviewed case reports differed in MSC concentration, regimes of MSC dosing and their origin. MSC therapy was well tolerated and the clinical effect was positive across the case reports, apart from in a report by Tao and colleagues, who noted that their patient required lung transplantation and died due to complications of transplant rejection [83,84,85,86,87,88,89,90,91,92,93]. The majority of research groups used human umbilical cord-mesenchymal stem cells (UC-MSCs) (seven case reports), while two groups used human menstrual blood-derived MSCs and two used bone marrow-derived MSCs. In all cases, the preferred route of administration was intravenous, with one report by Zengin and colleagues of intratracheal delivery concomitant to intravenous route (Table 1). Six clinical trials (phases 1 and 2) were also published enrolling a total of 239 patients. Across all studies, MSC therapy was applied intravenously and was deemed safe with a positive effect on clinical outcome based on the immunomodulatory effect of MSC therapy (Table 2).

Shi et al. conducted a phase 2 trial using UC-MSC to treat severe COVID-19 patients with lung damage. The study was randomized, double-blinded and placebo-controlled, with 65 patients in the UC-MSC group and 35 in the control group. Patients were given either UC-MSCs (4 × 10^7^/kg) or a placebo on days 0, 3 and 6. The results showed that infusion of UC-MSC significantly increased the resolution of lung solid component lesions, as determined by CT-imaging, compared with placebo on day 28 from baseline. To compare the restoration of lung function, a 6-min walk test (6-MWT) was performed and the results showed longer walking distance in the MSC group than in the placebo group. Furthermore, the incidence of adverse events in the MSC group and the placebo group was similar, and all adverse events were found to be unrelated to UC-MSC administration [95]. The same group previously reported a successful nonrandomized phase 1 clinical trial in which there were no serious adverse events associated with UC-MSC treatment. Facial flushing and fever were reported in two patients and another patient had transient hypoxia 12 h after MSC administration. The trial was conducted on 18 hospitalized patients with moderate and severe forms of the disease, out of whom nine received three cycles of intravenous infusion of UC-MSCs on days 0, 3 and 6, while the other nine patients were assigned to the control group [94].

Another group of authors used human menstrual blood-derived MSCs to treat severe and critically ill patients. The experimental group consisted of 26 patients who received MSC infusions (day 1, 3 and 5) and concomitant medications, while 18 patients in the control group received only concomitant medication. The results showed that mortality in the experimental group was significantly lower (7.69%) compared with the control group (33.33%). Furthermore, significant improvement in dyspnea was observed following MSC infusions in comparison with the control group. After adjustment for gender and age, the results suggested that MSC transplantation increased survival more for critically ill patients than for severe patients. Additionally, 5.8 days shorter time to recovery in the MSC group was observed, reaching statistical significance. The safety of the treatment was measured by the frequency of treatment-related adverse events (AEs). The results showed a statistically similar frequency of each AE, except for the AE related to high blood pressure, which was more common in the control group [96].

Similar results were seen in a double-blind, phase 1/2a randomized controlled trial which used umbilical cord MSCs in hospitalized patients suffering from ARDS secondary to COVID-19. Two intravenous infusions of UC-MSC were given to 12 patients (at days 0 and 3), while 12 patients in the control group received only vehicle solution containing human serum albumin and heparin. At 31 days after the first infusion, patient survival was significantly improved in the UC-MSC group compared with the control group: 10 out of 11 patients (91%) vs 5 out of 12 patients (42%), (*p* = 0.015) respectively. One of the patients in the UC-MSC group died due to failed endotracheal intubation; therefore, the authors censored data analyses for this subject at the time of failed endotracheal intubation. An increased risk of serious adverse events (SAEs) was reported in the control group as well as prolonged time to recovery. These findings were also correlated with a significant reduction in inflammatory markers (GM-CSF, IFN-γ, IL-5, IL-6, IL-7, TNF-α, TNF-β, PDGF-BB, and RANTES) when measured 6 days after UC-MSC treatment initiation. UC-MSC treatment did not lead to an increase in prespecified infusion-associated adverse events; therefore, it was deemed to be safe. Furthermore, the control group had an increased risk of SAEs compared with the UC-MSC group [97].

Another study also documented a significant reduction in proinflammatory mediators following the administration of UC-MSCs. A total of 12 patients were given the UC-MSC treatment and 29 patients formed the control group. Antivirals and glucocorticoids were administered to both groups before the trial. Compared with the control group, CRP and IL-6 levels were significantly decreased from day 3 of stem cell infusion in the UC-MSC group. Time to clinical improvement in the UC-MSC group was shorter, and chest CT scans indicated that patients in that group showed reduced lung inflammation compared with the control group. Although the differences in mortality between the groups did not reach statistical significance, improvement after UC-MSC application was visible [98].

A case series by Hashemian et al. of COVID-19 patients who developed ARDS reported significant relief of dyspnea and improvement in SpO2 in 5 out of 11 patients, 24–48 h after the first infusion of the umbilical cord or placental MSCs. Proinflammatory biomarkers (IL-8, TNF-α, CRP) decreased significantly in the first five days following the MSC infusions and the treatment was well tolerated, as no adverse events were observed that could be directly attributed to the procedure [99].

Another phase 1 clinical trial was conducted with the aim of assessing the safety and efficacy of intravenous MSC treatment. Five patients with severe COVID-19 were treated with Wharton’s jelly-derived MSCs, given in three intravenous injections three days apart. Monitoring was done on days 0, 3, 6 and 14. No serious complication associated with MSC application was observed except a slight postinjection headache in one of the patients which resolved after 30 min. Flow cytometry analysis was performed before and after MSC application and showed an upward trend of CD4 and CD8 markers after the MSC treatment, which could indicate an improvement in the lymphocyte population. Reduction of proinflammatory cytokines (IL-6, TNF-α, TGF-β1, IFN-γ) was observed after the treatment. MSC treatment was found to be safe and well-tolerated by patients. Further studies need to be conducted using control and treatment groups with increased sample sizes in order to draw conclusions about the efficacy of the treatment [100].

In a proof of concept study, 13 patients suffering from severe COVID-19 received adipose tissue-derived MSCs. All patients were mechanically ventilated before the first MSC administration. Ten patients received two doses (the second dose administered a median of three days after the first one), two patients received a single dose (these patients improved after the first dose, and therefore, were not given further doses) and one patient received three doses (following initial improvement, the patient worsened and was given the third dose). All of the patients were administered corticosteroids concomitantly to MSC treatment. The first dose of MSCs was administered at a median of seven days after initiation of mechanical ventilation. Median follow-up was 16 days after the first dose. Nine of the patients showed clinical improvement in the follow-up period. Seven patients were extubated, four remained intubated (two of which required extracorporeal mechanical oxygenation at the end of the follow-up period) while two patients died (one due to gastrointestinal bleeding not associated with MSC infusions). Furthermore, a decrease in inflammatory parameters (C-reactive protein, IL-6, ferritin, LDH and D-dimer) was noticed, as well as an increase in lymphocyte count. Changes in the aforementioned parameters were particularly noticeable in patients who showed clinical improvement. Interestingly, the authors observed that extubated patients had received MSC treatment earlier than those that were not extubated, which could indicate that application of MSC treatment early after intubation might improve the outcome [101].

A pilot trial of intravenous infusion of umbilical cord MSCs was conducted by Feng and colleagues on 16 severe and critical patients suffering from COVID-19. UC-MCSs were administered in four doses and the primary outcome of the study was oxygenation index on day 14 after the first UC-MSCs administration. The results indicated that the oxygenation index was improved after UC-MSCs transplantation. However, eight patients missed the arterial blood-gas analysis on day 14. The secondary outcome of the trial was to assess mortality on day 28, total length of hospital stay, radiological presentations on days 7, 14 and 28, inflammatory biomarkers on days 7, 14 and 28, and lymphocyte and its subsets count on days 7, 14 and 28. The mortality on day 28 was 6.25% (only one deceased patient), and there was no statistical significance in mortality between severe and critically severe patients. All patients showed improvement in the radiological appearance of the lungs compared to baseline. Although white blood cell count was similar in each follow-up, lymphocyte count showed recovery after UC-MSC transplantation. Furthermore, a decrease in proinflammatory cytokines count was observed. UC-MSC transplantation was considered safe, as no acute infusion-related or allergic reactions were documented [102].

## 5. Conclusions

COVID-19 still presents a challenge for modern medicine, as a significant proportion of patients present with severe clinical symptoms, and are often in critical conditions that end in death. A potential new treatment for these patients is the systemic application of MSCs. The available literature and reviewed clinical studies all reported favorable safety and beneficial clinical effects. However, there are currently no phase 3 clinical trials available that could confirm these findings in a broader patient cohort. Vaccination efforts worldwide could potentially end the pandemic, but the lessons learned will most certainly be translated in other fields of medicine. The effort of developing a potent immunomodulatory therapeutic option that is broadly available, safe and has been properly evaluated is promising for the treatment of a wide variety of infective, autoimmune and degenerative diseases.

## Figures and Tables

**Table 1:** Case reports of MSC treatment of COVID-19.

Author	Origin of MSCs Delivered	MSC Dosing	Method of Delivery
Zhang et al. [83]	umbilical cord	10^6^/kg single dose	iv
Tang et al. (2 cases) [84]	menstrual blood	10^6^/kg in 3 doses	iv
Liang et al. [85]	umbilical cord	5 × 10^7^/kg in 3 doses	iv
Peng et al. [86]	umbilical cord	10^6^/kg in 3 doses	iv
Tao et al. [87]	umbilical cord	1.5 × 10^6^/kg in 5 doses	iv
Zengin et al. [88]	umbilical cord	0.7 × 10^6^/kg in 2 doses iv	iv
		0.3 × 10^6^/kg in 2 doses it	it
Zhu et al. [89]	umbilical cord	10^6^/kg single dose	iv
Rich et al. [90]	bone marrow	10^6^/kg single dose	iv
Lu et al. [91]	menstrual blood	3000, 2000, 3000 units per dose	iv
Senegaglia et al. [92]	umbilical cord	5 × 10^5^/kg in 3 doses	iv
Primorac et al. [93]	bone marrow	10^6^/kg in 3 doses	iv

Iv—intravenous; it—intratracheal.

**Table 2:** Clinical trials of MSC treatment of COVID-19.

Author	Number of Participants (MSC-Control/Placebo)	Origin of MSCs Delivered	MSC Dosing
Meng et al. [94]	18 (9-9)	umbilical cord	3 × 10^7^ in 3 doses
Shi et al. [95]	100 (65-35)	umbilical cord	4 × 10^7^ in 3 doses
Xu et al. [96]	44 (26-18)	menstrual blood	3 × 10^7^ in 3 doses
Lanzoni et al. [97]	24 (12-12)	umbilical cord	100 ± 20 × 10^6^ in 2 doses
Shu et al. [98]	41 (12-29)	umbilical cord	2 × 10^6^/kg
Hashemian et al. [99]	11	umbilical cord (6 patients) or placental (5 patients)	200 × 10^6^ in 2 doses
Saleh et al. [100]	5	Wharton’s jelly	150 × 10^6^ in 3 doses
Sanchez-Gujio et al. [101]	13	adipose tissue	0.96 × 10^6^/kg in 1–3 doses
Feng et al. [102]	16	umbilical cord	10^8^ in 4 doses

## Data Availability

Not applicable.
